# Annotations

**Published:** 1886-10-23

**Authors:** 


### Annotations.

The Metropolitan Local Press.

There is no class of institutions in which the press shows greater interest than the hospitals. At the busiest season of the year an appeal from the sorely pressed hospital managers to the editorial compassion seldom fails to secure the desired response. There are, however, circumstances connected with the press which should be borne in mind by hospital managers who appeal for editorial aid. In the first place due care should be taken to send any paragraph or letter intended for publication in proper form. We once received a paragraph from a hospital secretary, about Christmas-time, in which we were asked "to gratefully acknowledge the receipt of a certain number of brace of pheasants from an illustrious personage, which would greatly assist the committee to meet the heavy financial depression from which their institution was suffering at the time." On the other hand the press sometimes has its whims. For instance, in June last, the new departure taken by the Hospital Sunday Fund, and known as the Hospitals Week, which was made a success by the aid of the daily press, was practically boycotted by the Metropolitan Local Press, with a few notable exceptions. One of these exceptions which deserves to be specially mentioned, was the action taken by the *Kentish Mercury*, which has probably a larger circulation, and greater influence, than any other single local paper published in the Metropolis. It devoted more than one leader to the movement, and to its efforts in no small degree is due the large measure of success which attended this movement in south-east London. Mr. James Watson, the editor of the *Kentish Mercury*, is well known from the fact that no philanthropic movement worthy of public support fails to win the help of his paper and his presence. Now that London has been divided into Parliamentary boroughs, it would be an immense advantage to the metropolitan institutions, as well as to the local press, if a sound public spirit could be aroused in each district which returns a member to the House of Commons. The metropolitan local press have had scant support and encouragement in the past, but the new electoral system ought to ensure an awakening of public spirit which should eventually make the local press in the Metropolis as influential and as ably conducted as any newspapers to be met with elsewhere throughout the country.

Advertising Doctors and Secret Remedies.

It is a well-known fact that among those who advertise certain specific but secret cures for consumption, cancer, and other fatal diseases, there are some legally qualified medical men. These men, by virtue of their legal qualifications, probably obtain a larger and more remunerative sale for their nostrums than the ordinary unqualified "quack" doctor. It is not, however, so well known that legally qualified men, on obtaining their diplomas, make oath that they will not in any way whatsoever make use of any "secret" remedy in the treatment of disease; but that, if they themselves discover any previously unknown remedy, or if any secret remedy be made known to them by any means, they will, with all reasonable promptitude, communicate their knowledge to the medical profession at large. The reason for such a professional adjuration is as obvious as it is benevolent. When, therefore, a legally qualified medical man professes to know and to use remedies unknown to other medical men, he, by that very profession, proclaims himself not only false to his oath but a wilful hider of secrets, the knowledge of which might save thousands of persons from prolonged suffering and untimely death. Whether such a person is likely to be an honest practitioner or a greedy knave the public must judge. All persons who advertise secret cures should be avoided as dangerous, whether legally qualified or not.

A Just Complaint.

Mr. W. C. Callaghan, of West Ham, has justly complained of the want of consideration which results from an absence of system in arranging for the accommodation and reception of those who attend the hospitals. It appears that his wife went to the Children's Hospital in Great Ormond Street, on the 5th inst., where she found that there was not sufficient sitting accommodation for those in attendance, and so had to stand with her child for two hours. At the end of this time it was intimated that the patients admitted within the hospital were so numerous as to render it impossible for the doctor to see them all in one day. In consequence Mrs. Callaghan, who had come all the way from West Ham, and who was no doubt full of tender anxiety for the sick child who accompanied her, had to return home without having seen the doctor, obtained relief, or even a word of consolation and comfort. A great outcry has justly been raised against the thoughtlessness and cruelty which permits young women in shops to be kept standing for hours throughout their day's work. How much more necessary then should it be to make provision at a hospital, and especially at a children's hospital, which must render it impossible for avoidable suffering and disappointment to arise to those who seek relief through its agency? Mr. Callaghan makes an excellent suggestion, which we hope will be widely adopted. He points out that the experience of each hospital ought to enable the officials to know approximately what number of patients the doctors will be able to attend to each day. If this were done, and so soon as the maximum number of patients had been admitted, the doors were to be closed, and a notice affixed thereto to the effect that no more patients could be admitted that day, the time of the patients would not be needlessly wasted, and much preventable suffering would be saved to children and adults alike.

**Hospitals and Dispensers.**

"In small proportions we just beauties see,

And in short measures life may perfect be."

There is no more useful or indispensable hospital official than the dispenser. He it is who has to compound all the drugs, to prepare the various mixtures and to supervise, and from time to time to suggest additions to the hospital pharmacopœia or drug directory. Out-patients know that the dispenser has it in his power to greatly facilitate or retard their comfort by the arrangements he makes for the expeditious supply of the preparations ordered for them by the physicians and surgeons. At some hospitals the dispenser becomes almost necessarily the friend and confidant of the patients, because he has to make certain inquiries, and to afford them the opportunity of contributing, if they are able, to the funds of the hospital. To the hospital managers the capable dispenser is, or ought to be, invaluable. If he is up to his work and takes a genuine interest in it, then the institution which he serves will be provided with the newest and latest remedies, and with only the best preparations and drugs, which will be procured at the lowest possible price. In a position of more or less subordination, and hidden away behind the high walls of his dispensary, where he is only accessible through the port-holes of delivery and supply, the outside world, and for that matter the hospital world, too, is often unconscious, or at least forgetful, of the excellent service this officer renders to humanity and charity in the course of his work. Nevertheless, and possibly because of his compulsory retirement, the dispenser is undoubtedly a person of no little importance in a hospital, and the assistance he is able to render to the managers in the matter of supplies must seriously affect the annual expenditure upon drugs and medical appliances, whilst the purity and freshness of the former are essential to the speedy recovery of the patients, and so tend to make him the means of dispensing the funds subscribed to hospitals in the wisest and best way, and to an extent little dreamt of by the uninitiated and unthoughtful.

